# How hepatitis C virus modifies the immunological profile of Sjögren syndrome: analysis of 783 patients

**DOI:** 10.1186/s13075-015-0766-3

**Published:** 2015-09-10

**Authors:** Pilar Brito-Zerón, Hoda Gheitasi, Soledad Retamozo, Albert Bové, María Londoño, Jose-Maria Sánchez-Tapias, Miguel Caballero, Belchin Kostov, Xavier Forns, Srini V. Kaveri, Manuel Ramos-Casals

**Affiliations:** Josep Font Autoimmune Diseases Laboratory, CELLEX, Institut d’Investigacions Biomèdiques August Pi i Sunyer (IDIBAPS), C/ Villarroel 170, Barcelona, 08036 Spain; Department of Autoimmune Diseases, Hospital Clínic, University of Barcelona, C/Villarroel, 170, 08036 Barcelona, Spain; Viral Hepatitis Unit, Liver Unit, CIBERehd, Hospital Clínic, University of Barcelona, IDIBAPS, C/ Villarroel 170, Barcelona, 08036 Spain; ENT Department, Hospital Clínic, University of Barcelona, IDIBAPS, C/ Villarroel 170, Barcelona, 08036 Spain; Primary Care Research Group, IDIBAPS, Primary Care Centre Les Corts, CAPSE, Mejia Lequerica, s / n, Barcelona, 08028 Spain; Immunopathology and Therapeutic Immunointervention, Centre de Recherche des Cordeliers, INSERM, 15 Rue de l’Ecole de Medecine, Paris, F-75006 France

## Abstract

**Introduction:**

We conducted a study to analyze how infection by hepatitis C virus (HCV) may influence the immunological serum pattern of patients with Sjögren syndrome (SS).

**Methods:**

Since 1994, we have tested serum HCV-IgG antibodies in 783 patients with SS diagnosed according to the 1993 European classification criteria. The immunological profile at diagnosis was compared according to the presence or absence of HCV.

**Results:**

Of the 783 patients with SS, 105 (13.4 %) tested positive for HCV-IgG antibodies (88 females, 17 males, mean age at SS diagnosis: 62.9 years). Multivariate analysis showed that patients with SS-HCV had a higher mean age and a higher frequency of low C3/C4 levels, cryoglobulins, and hematological neoplasia compared with patients without HCV. The frequency of anti-La antibodies compared with anti-Ro antibodies was higher in patients with SS-HCV (17 % vs. 15 %) and lower in patients without HCV infection (30 % vs. 43 %). The frequency of concomitant detection of the three main cryoglobulin-related markers (cryoglobulins, rheumatoid factor activity, and C4 consumption) was threefold higher in patients with SS-HCV compared with patients without HCV. SS-HCV patients with genotype 1b showed the highest frequencies of immunological abnormalities related to cryoglobulins and the lowest frequencies of anti-Ro/La antibodies.

**Conclusions:**

We found HCV infection in 13 % of a large series of Spanish patients with SS. The HCV-driven autoimmune response was characterized by a lower frequency of anti-Ro/La antibodies, an abnormal predominance of anti-La among anti-Ro antibodies, and a higher frequency of cryoglobulinemic-related immunological markers in comparison with patients without HCV infection. This immunological pattern may contribute to the poor outcomes found in patients with SS-HCV.

## Introduction

Sjögren syndrome (SS) is a systemic autoimmune disease that mainly affects the exocrine glands. This leads to dryness of the main mucosal surfaces, such as the mouth, eyes, nose, pharynx, larynx, and vagina [1]. The disease overwhelmingly affects middle-aged women but may also affect children, men, and older patients. The clinical spectrum of SS extends from dryness affecting the main mucosal surfaces to systemic involvement (extraglandular manifestations). SS may be a serious disease with excess mortality, which is related mainly to systemic involvement and hematological cancer [2].

In the etiopathogenesis of SS, a specific combination of individual genetic predisposition (intrinsic factors) and environmental agents (extrinsic factors) may be central to the development of the disease [3]. Viruses have always been considered one of the main exogenous culprits implicated in the etiopathogenesis of SS, and the hepatitis C virus (HCV) is a principal candidate [4]. In the last 15 years, several experimental, virological, and clinical studies have shown a close association between HCV and SS [5], suggesting that there may be shared immune-mediated etiopathogenic mechanisms. It sounds reasonable to investigate the role of the human ribonucleoproteins among these mechanisms. Human La protein is an essential factor in the biology of both coding and non-coding RNAs, is one of the principal autoantigens implicated in the etiopathogenesis of SS, and has been shown to play a key role in the initiation of HCV translation [6]. It could be hypothesized that patients carrying antibodies against Ro/La ribonucleoproteins are protected against chronic HCV infection.

The study of a large cohort of SS patients who were tested for HCV infection may help characterize the immunological profile of SS according to the presence or absence of HCV and reveal possible relationships between the main virological HCV features and the immunological expression of a systemic autoimmune disease characterized by an autoimmune response against human ribonucleoproteins, some of which have also been implicated in the translation and replication of HCV.

## Methods

Since 1994, we have tested 783 consecutive patients with primary SS diagnosed according to the 1993 European classification criteria for serum HCV-IgG antibodies [7]; patients with concomitant systemic autoimmune diseases other than SS were excluded. Fulfillment of the 2002 American-European criteria [8] and the preliminary 2012 American College of Radiology (ACR) criteria [9] was retrospectively evaluated: 470 (60 %) fulfilled the 2002 criteria, and 499 (64 %) the preliminary 2012 criteria (29 patients fulfilled the 2012 criteria but not the 2002 criteria since they had positive rheumatoid factor (RF) and antinuclear antibodies (ANA) titers above 320, but with negative Ro/La antibodies/salivary gland biopsy).

HCV infection was defined as a positive serological result for serum HCV antibodies in at least two determinations. Anti-HCV antibodies were detected by second-generation enzyme-linked immunosorbent assay (ELISA) between 1994 and 1998 and third-generation ELISA since 1998; all patients who tested positive for the second-generation ELISA were re-tested with the third-generation test. Serum HCV-RNA was detected by polymerase chain reaction, viral load by real-time polymerase chain reaction (COBAS TaqMan HCV Test, Roche Diagnostics, Manheim, Germany), and HCV genotype by restriction fragment-length polymorphism of the 5′ non-coding region of the HCV genome, as previously described [10]. Virological studies (serum HCV-RNA detection, viral load, and genotype) were carried out according to clinical reasons and were not available in all patients. SS patients who tested HCV antibody-positive but HCV-RNA-negative were considered as having a past HCV infection that was resolved, whereas those who tested positive for serum HCV-RNA were considered currently chronic HCV-infected.

A protocol form was used to retrospectively record the main characteristics of patients, including sex, age at diagnosis of SS (defined as the age when the patient fulfilled the current classification criteria), diagnostic tests for SS (ocular tests, salivary scintigraphy, salivary gland biopsy defined according to the recommendations of the European Community Study Group) [7], virological features (serum HCV-IgG, serum HCV-RNA, maximum viral load in the absence of anti-HCV therapies, HCV genotype), and adverse outcomes (neoplasia, death) until the last visit or death. The study was approved by the Ethics Committee of the Hospital Clinic of Barcelona (Spain), and the study design conformed to current Spanish ethical standards. Owing to the retrospective, observational, and anonymous nature of the study, informed patient consent was not required.

### Immunological studies

Immunological tests were made by using commercial techniques standardly used in the Spanish public health-care system, including ANA (indirect immunofluorescence using mouse liver and Hep-2 cells as substrate), precipitating antibodies to extractable nuclear antigens (Ro/SS-A, La/SS-B, U1-snRNP, and Sm; enzyme-linked immunoassay), and RF (nephelometry). Complement was measured by determination of C3 and C4 levels by nephelometry (BNII nephelometer; Dade Behring, Manburg, Germany). Serum cryoglobulins were measured after centrifugation. Blood samples were obtained and maintained at 37 °C for 30 min before separation. Serum was prepared by centrifuging at 37 °C for 10 min at 2500 revolutions per minute. Fresh centrifuged serum was incubated at 4 °C for 7 days after collection and examined for cryoprecipitation. Cryoglobulins were further analyzed by immunofixation when more than 5 % of cryoprecipitate was available. Serum monoclonal immunoglobulins were analyzed by immunofixation electrophoresis on agarose gels with specific antisera to IgG, IgM, IgA, and κ and λ chains at diagnosis and every year during the follow-up. Immunofixation was performed by using a Helena Immunofixation Agarose Kit (Helena Laboratories, Beaumont, TX, USA) in accordance with the instructions of the manufacturer.

### Statistical analysis

Descriptive data are presented as mean and standard deviation for continuous variables and as number and percentage for categorical variables. Qualitative differences were analyzed by using the chi-squared and Fisher’s exact tests. When several independent variables appeared to be statistically significant in the univariate analysis, logistic regression was made in a multivariate analysis to rule out possible confounding variables. To compare quantitative parameters, the Student’s *t* test was used in large samples of similar variance, and the non-parametric Mann–Whitney *U* test was used for small samples. A *P* value of less than 0.05 indicated statistical significance. The analysis was carried out by using the 18.0 SPSS program (SPSS, Chicago, IL, USA).

## Results

### Prevalence of HCV infection

Of the 783 patients with SS, 105 (13.4 %) tested positive for HCV-IgG antibodies (88 females and 17 males, with a mean age at SS diagnosis of 62.9 years). The prevalence varied according to the SS criteria fulfilled: 8 % (38/470) in patients fulfilling the 2002 criteria, 10 % (51/499) in patients fulfilling the 2012 ACR criteria, and 19 % (54/284) in patients fulfilling only the 1993 criteria; the highest percentage was found in the 29 patients who fulfilled the 2012 criteria but not the 2002 criteria (13 patients were HCV-positive, 45 %).

### Comparison between HCV-IgG-positive and -negative patients

Table 1 summarizes the main features of patients according to the presence or absence of serum HCV-IgG antibodies. In the univariate analysis, patients with SS-HCV had a higher mean age (63 vs. 56 years, *P* < 0.001), were more frequently male (16 % vs.7 %, *P*=0.003), less frequently fulfilled the 2002 SS criteria (26 % vs. 64 %, *P* < 0.001), and had a higher frequency of RF (56 % vs. 40 %, *P*=0.004), low C3 levels (36 % vs. 11 %, *P* < 0.001), low C4 levels (48 % vs. 7 %, *P* < 0.001), serum monoclonal gammopathy (47 % vs. 17 %, *P* < 0.001), and cryoglobulinemia (61 % vs. 7 %, *P* < 0.001); a lower frequency of ANA (76 % vs. 84 %, *P*=0.05), anti-Ro (15 % vs. 43 %, *P* < 0.001), and anti-La (17 % vs. 30 %, *P* < 0.001); and a higher frequency of adverse outcomes, including hematological neoplasia (9 % vs. 4 %, *P*=0.04), neoplasia (20 % vs. 8 %, *P* < 0.001), and death (33 % vs. 8 %, *P* < 0.001) compared with patients without HCV infection; age, anti-Ro antibodies, low C3 levels, low C4 levels, cryoglobulins, and hematological neoplasia were significant independent variables in the multivariate model.

**Table 1:** Main SS-related features of patients according to the presence or absence of serum HCV-IgG antibodies

	Negative HCV-IgG	Positive HCV-IgG	Bilateral *P* value
	N = 678	N = 105	
Mean age, years	56.36 ± 14.83	62.93 ± 11.86	<0.001*
Sex, male	45 (7 %)	17 (16 %)	**0.003**
Dry mouth	661 (98 %)	103 (98 %)	1
Dry eye	661 (98 %)	104 (99 %)	0.493
Altered ocular tests	559/609 (92 %)	82/88 (93 %)	0.834
Altered parotid scintigraphy	486/554 (88 %)	45/54 (83 %)	0.389
Positive salivary gland biopsy	198/309 (64 %)	21/30 (70 %)	0.556
Criteria SS			
- 1993 only	230 (34 %)	54 (51 %)	**<0.001**
- 2002	432 (64 %)	38 (26 %)	
- ACR only	16 (2 %)	13 (12 %)	
Antinuclear antibody^+^	568/676 (84 %)	79/104 (76 %)	**0.05**
Rheumatoid factor^+^	267/663 (40 %)	57/102 (56 %)	**0.004**
Anti-Ro/SS-A^+^	292/676 (43 %)	16/103 (15 %)	<0.001*
Anti-La/SS-B^+^	200/676 (30 %)	17/103 (17 %)	**0.006**
Monoclonal gammopathy	85/492 (17 %)	35/75 (47 %)	**<0.001**
Type of monoclonal band			
- mIgA	9 (11 %)	1 (3 %)	**0.047**
- mIgG	47 (55 %)	13 (37 %)	
- mIgM	18 (21 %)	15 (43 %)	
- Free chains	11 (13 %)	6 (17 %)	
Type of monoclonal light chain			
- Kappa:lambda	50:35	19:16	0.65
Cryoglobulin^+^	41/626 (7 %)	63/104 (61 %)	<0.001*
Low C3 levels, <0.82 g/l	70/660 (11 %)	37/103 (36 %)	<0.001*
Low C4 levels, <0.11 g/l	45/660 (7 %)	49/103 (48 %)	<0.001*
Hematological neoplasia	26 (4 %)	9 (9 %)	0.04*
Neoplasia	54 (8 %)	21 (20 %)	<0.001*
Death	52 (8 %)	35 (33 %)	**<0.001**

*Statistically significant in the multivariate model.

*SS* Sjögren syndrome, *HCV* hepatitis C virus, *IgG* immunoglobulin G, *ACR* American College of Rheumatology, *SS-A* Sjögren syndrome A antigen, *SS-B* Sjögren syndrome B antigen, *mIg* circulating monoclonal immunoglobulin, *C3* complement component 3, *C4* complement component 4Bold numbers: statistically-significant differences in the univariate analysis (p<0.05)

### Characteristics of SS patients with past HCV infection

Serum HCV-RNA determination was available in 77 (73 %) of the 105 SS patients who tested positive for HCV-IgG antibodies. Seven (9 %) of the 77 patients were negative for serum HCV-RNA and were considered as having a resolved HCV infection. All seven patients were female, with a mean age of 66 years, and all had the same autoantibody profile (positive for ANA but negative for anti-Ro and anti-La antibodies). No statistically significant differences existed between patients with past HCV infection and those with chronic HCV infection (data not shown).

### Association between HCV genotypes and SS features

Genotype determination was available in 44 (42 %) patients with SS: 30 (70 %) patients had genotype 1b, eight (19 %) genotype 1a, and the remaining five (11 %) non-1 genotypes, including genotypes 2 (n=1), 3 (n=2), and 4 (n=2); in one sample, HCV was not genotypable by the molecular assay. Table 2 summarizes the main features of SS patients according to the main HCV genotypes. Patients with genotype 1b had the highest frequencies of immunological abnormalities related to cryoglobulins (cryoglobulinemia, monoclonal gammopathy, and RF) and the lowest frequencies of anti-Ro/La antibodies compared with patients with other genotypes.

**Table 2:** Main SS-related features of patients according to the HCV genotypes (1a, 1b, and non-1 genotypes)

	Genotype 1a	Genotype 1b	Non-1 genotypes	Bilateral *P* value
	N = 8	N = 30	N = 5	
Mean age, years	62.62 ± 12.62	64.93 ± 9.81	46.00 ± 8.15	**0.002**
Sex, male	0 (0 %)	8 (27 %)	2 (40 %)	0.182
Dry mouth	8 (100 %)	30 (100 %)	5 (100 %)	1.000
Dry eye	8 (100 %)	30 (100 %)	5 (100 %)	1.000
Altered ocular tests	5/6 (83 %)	23/24 (96 %)	4/5 (80 %)	0.381
Altered parotid scintigraphy	4/5 (80 %)	14/17 (82 %)	2/3 (67 %)	0.822
Positive salivary gland biopsy	5/5 (100 %)	3/5 (60 %)	0/2 (0 %)	**0.037**
Criteria SS				
- 1993 only	3 (37 %)	20 (67 %)	2 (40 %)	0.175
- 2002	5 (63 %)	7 (23 %)	3 (60 %)	
- ACR only	0 (0 %)	3 (10 %)	0 (0 %)	
Antinuclear antibody^+^	6 (75 %)	26 (87 %)	4 (80 %)	0.709
Rheumatoid factor^+^	3 (37 %)	16/29 (55 %)	1 (20 %)	0.284
Anti-Ro/SS-A^+^	2 (25 %)	3 (10 %)	2 (40 %)	0.185
Anti-La/SS-B^+^	1 (12 %)	5 (17 %)	2 (40 %)	0.410
Monoclonal gammopathy	3 (37 %)	13/26 (50 %)	0/1 (0 %)	0.535
Type of monoclonal band				
- mIgA/mIgG/mIgM/free chains	0/0/2/1	1/6/5/1	0/0/0/0	0.34
- Kappa:lambda light chains	1:2	6:7	0	1.000
Cryoglobulin^+^	4 (50 %)	20/29 (69 %)	0 (0 %)	**0.014**
Low C3 levels, <0.82 g/l	3 (37 %)	12 (40 %)	3 (60 %)	0.677
Low C4 levels, <0.11 g/l	4 (50 %)	13 (43 %)	2 (40 %)	0.926
Hematological neoplasia	1 (12 %)	3 (10 %)	1 (20 %)	0.809
Neoplasia	1 (12 %)	6 (20 %)	1 (20 %)	0.886
Death	4 (50 %)	9 (30 %)	0 (0 %)	0.161
Viral load > 5,000,000	2 (25 %)	11/28 (39 %)	0/5 (0 %)	0.095
Max viral load (log)	6.36 ± 0.39	6.13 ± 0.55	5.96 ± 0.38	0.350

*SS* Sjögren syndrome, *HCV* hepatitis C virus, *IgG* immunoglobulin G, *ACR* American College of Radiology, *SS-A* Sjögren syndrome A antigen, *SS-B* Sjögren syndrome B antigen, *mIg* circulating monoclonal immunoglobulin, *C3* complement component 3, *C4* complement component 4Bold numbers: statistically-significant differences in the univariate analysis (p<0.05)

### Influence of HCV on the SS-related autoantibody profile

Table 3 shows the distribution of the main SS-related immunological markers (ANA, RF, anti-Ro and anti-La antibodies, cryoglobulins, low C3/C4 levels) according to the presence or absence of HCV infection. A total of 232 patients with SS had positive ANA or RF (or both) in the absence of anti-Ro/La antibodies; SS patients without HCV had a higher frequency of isolated ANA and a lower frequency of isolated RF compared with patients with SS-HCV (65 % vs. 43 % and 4 % vs. 18 %, respectively, *P* <0.001). Anti-Ro/La antibodies were positive in 335 patients (190 had both autoantibodies, 118 only anti-Ro, and 27 only anti-La). Patients with SS-HCV had a lower frequency of isolated anti-Ro antibodies and a higher frequency of isolated anti-La antibodies compared with SS patients without HCV infection (36 % vs. 19 % and 24 % vs. 7 %, respectively, *P*=0.014). Patients with SS-HCV had a higher frequency of combined cryoglobulin-related markers (cryoglobulins, RF, and low C4) and a lower frequency of isolated cryoglobulins compared with SS patients without HCV infection (47 % vs. 18 % and 15 % vs. 34 %, respectively, *P*=0.019).

**Table 3:** Combination of the main SS-related immunological profiles (ANA/RF, Ro/La, and cryoglobulinemic-related markers) according to the presence or absence of serum HCV-IgG antibodies

ANA/RF combination	Negative HCV-IgG	Positive HCV-IgG	Bilateral *P* value
	N = 306	N = 74	
ANA and RF	94 (31 %)	29 (39 %)	<0.001
Isolated ANA	200 (65 %)	32 (43 %)	
Isolated RF	12 (4 %)	13 (18 %)	
Ro/La combination	Negative HCV-IgG	Positive HCV-IgG	Bilateral *P* value
	N = 314	N = 21	
Anti-Ro and anti-La antibodies	178 (57 %)	12 (57 %)	0.014
Isolated anti-Ro antibodies	114 (36 %)	4 (19 %)	
Isolated anti-La antibodies	22 (7 %)	5 (24 %)	
Cryoglobulinemic-related markers combination	Negative HCV-IgG	Positive HCV-IgG	Bilateral *P* value
	N = 38	N = 60	
Cryoglobulins+RF+low C4	7 (18 %)	28 (47 %)	0.019
Cryoglobulins+RF	12 (32 %)	13 (22 %)	
Cryoglobulins+low C4	6 (16 %)	10 (17 %)	
Isolated cryoglobulins	13 (34 %)	9 (15 %)	

*SS* Sjögren syndrome, *ANA* antinuclear antibodies, *RF* rheumatoid factor, *HCV* hepatitis C virus, *IgG* immunoglobulin G, *C4* complement component 4

### Comparison according to the presence of antibodies against the La autoantigen

Patients with SS-HCV had a higher frequency of anti-La antibodies than of anti-Ro antibodies (17 % vs. 15 %). Comparison of the main characteristics of patients with SS-HCV according to the presence or absence of anti-La antibodies showed no significant differences (data not shown). We also compared the main features of the SS patients carrying anti-La antibodies according to the presence or absence of HCV infection (Table 4). SS-HCV patients carrying La autoantibodies had a higher mean age (59.6 vs. 51 years, *P*=0.03), were more frequently male (23 % vs. 4 %, *P*=0.003), and had a higher frequency of low C3 levels (53 % vs. 10 %, *P* < 0.001), low C4 levels (65 % vs. 7 %, *P* < 0.001), serum monoclonal gammopathy (54 % vs. 20 %, *P*=0.01), and cryoglobulinemia (53 % vs. 10 %, *P* < 0.001); a predominance of lambda monoclonal gammopathies (71 % vs. 30 %, *P*=0.041); a lower frequency of anti-Ro (71 % vs. 89 %, *P*=0.044) and anti-Ro (15 % vs. 43 %, *P* < 0.001); and a higher frequency of death (41 % vs. 8 %, *P*=0.001) compared with HCV-negative SS patients carrying anti-La antibodies.

**Table 4:** Main SS-related features of the 17 SS-HCV patients with anti-La antibodies in comparison with the 200 SS patients with anti-La antibodies without HCV infection

	Anti-La^+^ SS patients with HCV infection	Anti-La^+^ SS patients with no HCV infection	Bilateral *P* value
	N = 17	N = 200	
Mean age, years	59.59 ± 11.87	51.04 ± 15.77	**0.03**
Sex, male	4 (23 %)	8 (4 %)	0.009*
Dry mouth	16 (94 %)	197 (99 %)	0.280
Dry eye	17 (100 %)	196 (98 %)	1.000
Altered ocular tests	15/16 (94 %)	176/187 (94 %)	1.000
Altered parotid scintigraphy	9/10 (90 %)	151/165 (91 %)	0.602
Positive salivary gland biopsy	3/3 (100 %)	55/61 (90 %)	1.000
Antinuclear antibody^+^	14 (82 %)	170/199 (85 %)	0.723
Rheumatoid factor^+^	13/17 (77 %)	112/191 (59 %)	0.199
Anti-Ro/SS-A^+^	12 (71 %)	178 (89 %)	**0.044**
Monoclonal gammopathy	7/13 (54 %)	33/166 (20 %)	**0.01**
Type of monoclonal band			
- mIgA	0 (0 %)	3 (9 %)	0.87
- mIgG	4 (57 %)	17 (52 %)	
- mIgM	2 (29 %)	9 (27 %)	
- Free chains	1 (14 %)	4 (12 %)	
Type of monoclonal light chain			
- Kappa:lambda	2:5	23:10	**0.041**
Cryoglobulin^+^	9 (53 %)	19/188 (10 %)	**<0.001**
Low C3 levels, <0.82 g/l	9 (53 %)	22/196 (11 %)	<0.001*
Low C4 levels, <0.11 g/l	11 (65 %)	14/196 (7 %)	<0.001*
Hematological neoplasia	0 (0 %)	7 (3.5 %)	1.000
Neoplasia	4 (23.5 %)	17 (8.5 %)	0.067
Death	7 (41 %)	16 (8 %)	**0.001**

*Statistically significant in the multivariate model

*SS* Sjögren syndrome, *HCV* hepatitis C virus, *SS-A* Sjögren syndrome A antigen, *mIg* circulating monoclonal immunoglobulin, *C3* complement component 3, *C4* complement component 4Bold numbers: statistically-significant differences in the univariate analysis (p<0.05)

## Discussion

HCV was identified as the main cause of the so-called non-A non-B viral hepatitis 25 years ago [11]. Later, a close link between chronic HCV infection and the development of autoimmune processes was reported, principally with two systemic autoimmune diseases: cryoglobulinemic vasculitis and SS [12]. A large number of studies have linked SS with HCV, including the common finding of focal sialadenitis in patients with HCV [13], the development of an SS-like exocrinopathy in transgenic mice carrying the HCV envelope genes [14], the infection and replication of HCV in epithelial cells from salivary glands of patients with SS or chronic sialadenitis [15], and the discovery of the key role of the La protein in facilitating the internal initiation of translation and replication of HCV-RNA [16]. These findings support a specific role for HCV in the etiopathogenesis of a subgroup of patients with diagnosed “primary” SS.

We found HCV infection in 13 % of a large series of Spanish patients who fulfilled the 1993 classification criteria for SS (8 % in patients fulfilling the more restrictive 2002 criteria). Various studies have analyzed the prevalence of chronic HCV infection in patients with primary SS, and most have found a higher prevalence than in the general population, although the results vary according to the geographic area. Studies from southern Europe describe a prevalence ranging from 10 % to 20 % (14 % using ELISA-3 and 5 % to 19 % using RIBA-2) [5]. In contrast, studies from Scandinavia and the USA have found no association between SS and HCV (prevalence of less than 1 %) and this is possibly due to the lower prevalence of HCV infection in these countries compared with the Mediterranean area [17]. We tested the largest SS population from a single center for HCV infection and found a prevalence 10-fold higher than that found in the general Spanish population [18], although it is possible that there may be a potential referral bias.

Demographically, SS-HCV was characterized by a comparatively reduced female-to-male ratio (5:1 vs. 14:1 in patients with primary SS) and an older age at SS diagnosis. The clinical phenotype of SS-HCV was indistinguishable from that of primary SS, and there were no significant differences in the prevalence of sicca features and the corresponding diagnostic tests. The sialotropism of HCV [4] may explain the close association with SS, including the results of salivary gland biopsies in patients with HCV. Experimental studies have reported that the envelope proteins of HCV may recruit lymphocytes in the salivary glands, leading to the formation of lymphocytic infiltrates, as occurs in primary SS (focal sialadenitis) [13–15]. De Vita et al. [19] first detected HCV in human salivary glands, and two additional studies [20, 21] have demonstrated the capability of the HCV to infect and replicate in the salivary gland tissue of HCV patients with sicca syndrome/SS. The reasons for the specific predilection of HCV for infecting exocrine gland tissue are unknown.

The autoantibody profile of patients with HCV-related SS is characterized by a higher frequency of RF and a lower frequency of Ro/La antibodies [5]. This immunological pattern influences the fulfillment of the new classification criteria proposed after the 1993 criteria, making fulfillment of the 2002 criteria (positivity for Ro/La is mandatory in the absence of a positive salivary gland result) more difficult than fulfillment of the 2012 criteria (these criteria allow the inclusion of patients with positive ANA/RF even in the absence of Ro/La antibodies). As the results of this study show, the prevalence of HCV infection in patients with SS may vary widely according to the set of criteria used to classify patients with SS.

In patients with SS, the HCV-driven autoimmune response is dominated principally by the presence of mixed cryoglobulins, reported in nearly two thirds of patients with SS-HCV (a ninefold higher prevalence with respect to patients with primary SS). Cryoglobulins play a predominant role in the global immunological pattern of these patients and is closely associated with positive RF, monoclonal gammopathy, and low C4 levels, whose frequencies (either isolated or in combination) were higher than those observed in patients without HCV. In addition, we found significant differences in the serum monoclonal expression (frequency and heterogeneity) of patients with SS according to the presence or absence of HCV infection. The prevalence of circulating mIgs in patients with SS-HCV was threefold higher than in patients without HCV, and mIgMκ, which was closely related to mixed cryoglobulinemia, was the most common type of circulating mIg, whereas in patients without HCV, mIgGk was the predominant circulating monoclonal band. We also found that SS-HCV patients with monoclonal gammopathy had a more restrictive monoclonal expression (overwhelmingly limited to either mIgMk or mIgG) compared with patients without HCV, who presented all types of monoclonal heavy and light chains. This suggests that HCV may play an important role in the clonal selection of specific B cells [22].

The lymphotropism of HCV links the virus not only to the synthesis of cryoglobulins but also to the development of lymphoma [23], and we found a higher frequency of hematological neoplasia in patients with SS-HCV compared with those without HCV. Lymphomagenesis in patients with HCV might be initiated by chronic stimulation of polyclonal B cells by the virus [24] and the compartmentalization of HCV quasispecies in blood mononuclear cells [25], with the posterior development of specific B-cell clonal expansions and pro-carcinogenic mutations [26, 27], which are similar to the etiopathogenic mechanisms of lymphoma development reported in primary SS [24, 28, 29]. Primary SS is the systemic autoimmune disease with the highest risk of lymphoma development [30]: we found that the combination of HCV and SS doubled the risk reported in patients with SS alone. Both SS and chronic HCV infection are characterized by underlying B-cell hyperactivity which predisposes to monoclonal B-cell selection [31]; therefore, SS-HCV may present one of the highest risks of overt B-cell lymphoma of all systemic autoimmune diseases.

Human La protein is known to be an essential host factor for the translation and replication of HCV RNA [32]. Translation of HCV is an essential step of viral replication and is mediated by an internal ribosome entry site, and the ribonucleoprotein La is a potent regulator for the enhancement of HCV replication [33]. We tested the hypothesis that patients carrying anti-La antibodies could be protected against chronic HCV infection. Unfortunately, the results suggest that serum anti-La antibodies do not play a significant protective role against chronification of HCV infection in patients with SS. None of the seven SS patients who had a resolved HCV infection (positive HCV-IgG with negative HCV-RNA) carried anti-La antibodies. In addition, we found no significant differences in the epidemiological, clinical, and immunological profile of La-positive patients according to the presence or absence of HCV infection.

We found an abnormal Ro/La immunological pattern according to the presence or absence of HCV infection. First, we found that the prevalence of anti-Ro/La antibodies was significantly reduced in patients with SS-HCV compared with patients without HCV. Second, we found more patients with SS-HCV were carrying La autoantibodies than those carrying anti-Ro autoantibodies, and this is in clear opposition to results found in SS patients without HCV. Third, we found that nearly one quarter of Ro/La^+^ SS-HCV patients carried isolated anti-La antibodies, an immunological pattern rarely reported in primary SS (<5 %). These findings suggest that, in patients with SS, the autoimmune response against the human La protein is significantly altered by the concomitant presence of HCV infection, an abnormal response probably related to the use of human ribonucleoproteins by the virus.

The study has some limitations related to the small number of patients included in some comparisons, such as the low number of patients with resolved HCV infection (only 7) or the low percentage of HCV patients with available viral genotyping in whom a salivary gland biopsy was carried out (only 12 out of 43 patients). In these comparisons, the statistically significant results should be taken with caution and require confirmation by studies including a large number of patients.

## Conclusions

In summary, we found HCV infection in 13 % of a large series of Spanish patients with SS. The HCV-driven autoimmune response in patients with SS-HCV is characterized by a lower frequency of autoantibodies against Ro and La human ribonucleoproteins, an abnormal predominant presence of anti-La among anti-Ro antibodies, and a higher frequency of cryoglobulinemic-related immunological markers (including positive RF) in comparison with SS patients without HCV. This immunological pattern influences the fulfillment of the SS classification criteria and may be related to the increased prevalence of hematological neoplasia that we found in patients with SS-HCV.

## Abbreviations

ACR: American College of Rheumatology
ANA: Antinuclear antibodies
C3: Complement component 3
C4: Complement component 4
ELISA: Enzyme-linked immunosorbent assay
HCV: Hepatitis C virus
HEp-2: Human epithelial type 2
IgA: Immunoglobulin A
IgG: Immunoglobulin G
IgM: Immunoglobulin M
mIg: Circulating monoclonal immunoglobulin
RF: Rheumatoid factor
Sm: Smith
SS: Sjögren syndrome
SS-A: Sjögren syndrome A antigen
SS-B: Sjögren syndrome B antigen
U1-snRNP: U1 small nuclear ribonucleoprotein particle
